# DNA damage-induced activation of ATM promotes β-TRCP-mediated ARID1A ubiquitination and destruction in gastric cancer cells

**DOI:** 10.1186/s12935-019-0878-y

**Published:** 2019-06-14

**Authors:** Zhou-hua Jiang, Tao Peng, Hai-long Qian, Cai-de Lu, Feng Qiu, Su-zhan Zhang

**Affiliations:** 1 Zhejiang University School of Medicine, Hangzhou, 310009 Zhejiang China; 2 Department of Gastrointestinal Surgery, Ningbo Medical Center, Li Huili Eastern Hospital, Ningbo, 315000 Zhejiang China; 3 Department of Surgical Oncology, The Second Affiliated Hospital, Zhejiang University School of Medicine, Hangzhou, 310009 Zhejiang China

**Keywords:** ARID1A, β-TRCP, Phosphodegron, DNA damage

## Abstract

**Background:**

AT-rich interactive domain-containing protein 1A (ARID1A) is a subunit of the mammary SWI/SNF chromatin remodeling complex and a tumor suppressor protein. The loss of ARID1A been observed in several types of human cancers and associated with poor patient prognosis. Previously, we have reported that ARID1A protein was rapidly ubiquitinated and destructed in gastric cancer cells during DNA damage response. However, the ubiquitin e3 ligase that mediated this process remains unclear.

**Materials and methods:**

The interaction between ARID1A and β-TRCP was verified by co-immunoprecipitation (Co-IP) assay. The degron site of ARID1A protein was analyzed by bioinformatics assay. Short hairpin RNAs (shRNAs) were used to knockdown (KD) gene expression.

**Results:**

Here we show that DNA damage promotes ARID1A ubiquitination and subsequent destruction via the ubiquitin E3 ligase complex SCFβ-TRCP. β-TRCP recognizes ARID1A through a canonical degron site (DSGXXS) after its phosphorylation in response to DNA damage. Notably, genetic inactivation of the Ataxia Telangiectasia Mutated (ATM) kinase impaired DNA damage-induced ARID1A destruction.

**Conclusions:**

Our studies provide a novel molecular mechanism for the negative regulation of ARID1A by β-TRCP and ATM in DNA damaged gastric cancer cells.

## Background

Recent genome-wide association studies (GWAS) have demonstrated that the AT-rich interactive domain 1A (ARID1A) gene is frequently mutated in a wide variety of cancer [1–3]. The majority of mutations of ARID1A are insertions/deletions, suggesting ARID1A is a tumor suppressor gene [4]. Indeed, ARID1A collaborates with p53 to regulate genes transcription and tumor growth in gynecologic cancers [5, 6]. ARID1A encodes a large nuclear protein and is a component of the switch/sucrose non-fermentable (SWI/SNF) complex by interacting with several other proteins including SMARCD3 [7]. SWI/SNF, is a master regulator of transcription factor action and enable gene transcription and/or repressing by promoting or preventing transcription factors to bind to promoters and/or enhancers and plays a critical role in DNA damage response, mitosis and genomic instability [8, 9].

Gastric cancer (GC) is the fourth most common cancer and the second leading cause of cancer death worldwide [10]. The raising of gastric cancer is known to be involve by multiple genetic and epigenetic alterations, which resulted in the aberrant regulation of many cancer-associated genes, which play critical role in diverse cellular processes [11, 12]. It has been shown that ARID1A was mutated and downregulated in GC and restoring ARID1A expression in gastric cancer cells significantly inhibited cell proliferation and colony formation [6, 13, 14]. However, how to regulate ARID1A itself is still not fully understood. Previously, we found that ARID1A was rapidly ubiquitinated and destructed in response to DNA damage and associated with both SKP1 and Cullin1 which are the components of Skp1-Cul1-F box protein (SCF) ubiquitin ligases [15].

There are 69 SCF ligases in human cells, and are distinguished by the exchangeable F box proteins that provide specificity for the SCF E3 ligases [16]. Phosphorylation on specific sites are requested for most SCF substrates which are recognized by F box proteins [17]. However, there are only 3 out of the entire F box proteins, including Skp2, β-TRCP, and Fbxw7 that have well-established substrates [18]. Human cells express two distinct β-TRCP proteins (β-TRCP1 and β-TRCP2), but with undistinguishable biochemical function, therefore we use the term β-TRCP to refer to both proteins [19].

The aim of this study is therefore to determine which F-box protein is involved in the degradation of ARID1A. By using an unbiased F-box proteins binding screen assay, we identified β-TRCP is the E3 ligase for ARID1A degradation and found that β-TRCP interacted and ubiquitinated ARID1A in a phosphorylation-dependent manner during DNA damage response.

## Materials and methods

### Cell culture

HEK293T cells and gastric cancer cells line NCI-N87 and AGS cells were cultured in Dulbecco’s modified Eagle’s medium (Invitrogen) supplemented with 10% fetal calf serum (Gibco BRL, Gaithersburg, MD). All these cells were cultured in a 5% CO_2_/95% air at 37 °C. DMSO, proteasome inhibitor MG132, cycloheximide (CHX), λ-ppase and VP16 (Etoposide) were purchased from sigma.

### Plasmids and transfection

ARID1A plasmid was purchased from Addgene (#39475). Flag-tagged F-box protein plasmids were gifts from Liu [20]. ARID1A and β-TRCP mutants were generated using QuickChange Site-Directed Mutagenesis Kit (Stratagene). All cDNAs were completely sequenced. The following shRNA-expression lentiviral plasmids were made in PLKO.1 and purchased from sigma, with the clone numbers indicated: β-TRCP (TRCN0000314899 and TRCN0000314972) and ATM (TRCN0000194969 and TRCN0000195732). All the transient transfections were performed with Lipofectamine 2000 (Invitrogen) according to the manufacturer's instructions.

### Western blotting

Protein extracts were loaded on 10–12% SDS-PAGE, electrophoresed, and transferred to nitrocellulose (NC) membrane. After blocking with 5% nonfat milk in PBS, the membranes were then incubated with the primary antibodies and followed by horseradish peroxidase (HRP)—linked secondary antibodies. The signals were detected by chemiluminescence phototope-HRP kit WBKLS0100 (Millipore, USA) according to manufacturer’s instructions. Antibodies were obtained from the following sources: anti-ARID1A (Santa Cruz Biotech, Santa Cruz, CA), anti-β-TRCP (D13F10) Rabbit mAb (Cell Signaling, Beverly, MA), anti-ATM (Santa Cruz Biotech, Santa Cruz, CA), anti-Flag M2 (Sigma, USA), anti-HA (Sigma, USA), anti-Cullin1 (Santa Cruz Biotech, Santa Cruz, CA), anti-SKP1 (Santa Cruz Biotech, Santa Cruz, CA) and anti-β-actin (Cell Signaling, Beverly, MA).

### Immunoprecipitation (IP)

Cells were lysed in 5 ml of lysis buffer (150 mM Tris–HCl pH 7.5, 150 mM NaCl, 0.5% Nonidet P40, and 50 mM PMSF) for 20 min at 4 °C and sonicated for 4 min. Lysates were cleared using centrifugation (13,000 rpm, 20 min), the supernatant was then subjected to IP with 15 μl anti-mouse IgG or HA antibody with 20 μl protein G beads (Sigma) overnight at 4 °C with gentle rotation. Beads containing immune complexes were washed with lysis buffer 6 times. Precipitates were denatured in 2× SDS buffer at 99 °C for 5 min. For Flag-tagged protein IP, the supernatant was then subjected to IP with 20 μl Flag M2 beads (Sigma) overnight at 4 °C with gentle rotation.

### CHX assay

To analyze protein half-life, cells were treated with CHX (25 μg/ml) for different durations followed by western blot assay.

### Statistical analysis

Values were shown as mean ± SEM. Statistical differences were determined by a Student t test. Statistical significance is displayed as *P < 0.05, **P < 0.01 or ***P < 0.001.

## Results

### β-TrCP associated with ARID1A

**Fig. 1:**
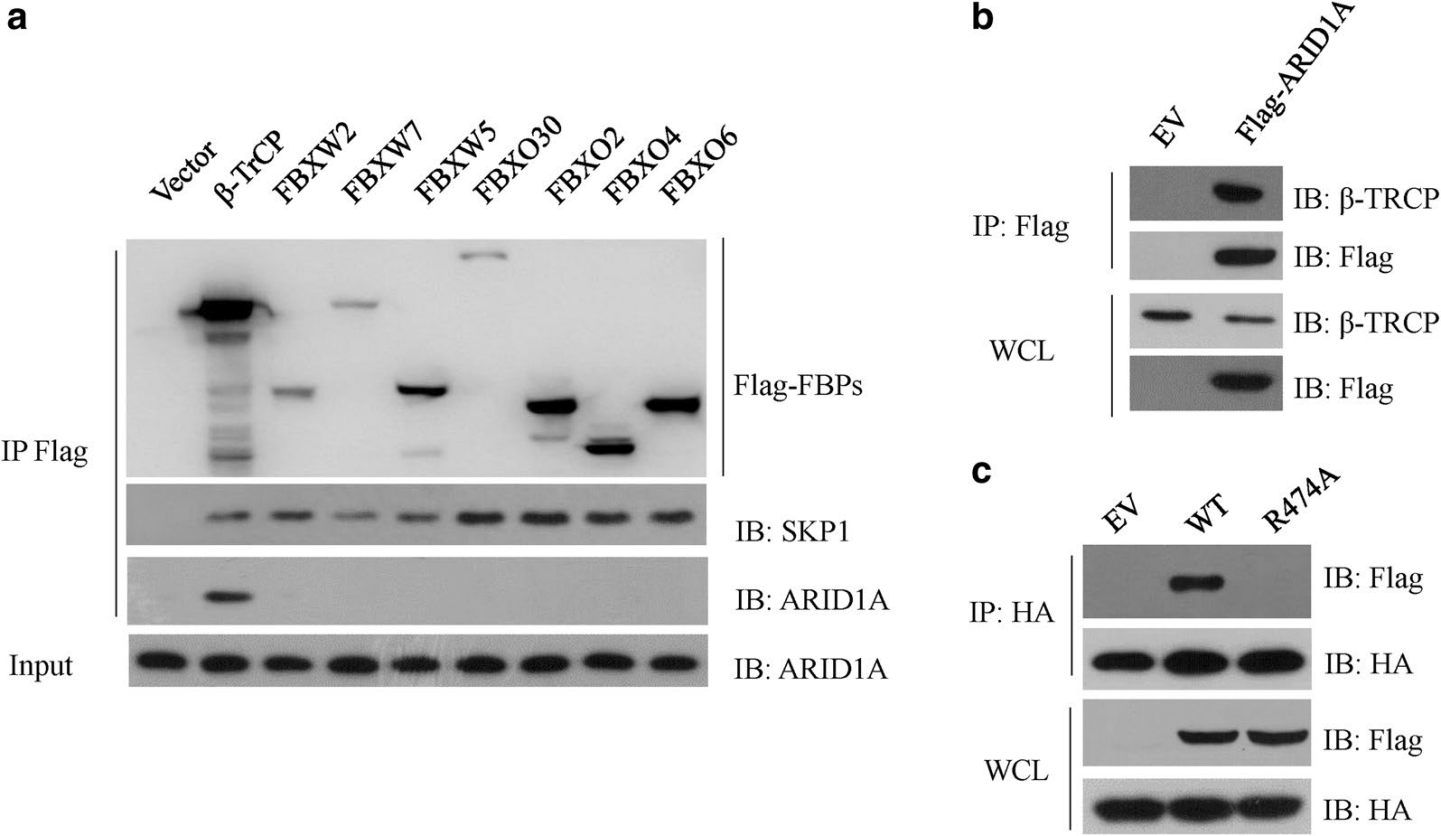
β-TrCP associated with ARID1A. **a** HEK293T cells were transfected with empty vector (EV) or the indicated FLAG-tagged F-box protein plasmids (FBPs). Cell extracts were immunoprecipitated (IP) with anti-FLAG M2 beads, and the immunocomplexes were subjected to western blot with the indicated antibodies. **b** HEK293T cells were transfected with Flag-ARID1A for 36 h, cell extracts were IP with anti-FLAG M2 beads, and the immunocomplexes were subjected to western blot with the indicated antibodies. **c** HEK293T cells were co-transfected with Flag-ARID1A and HA-β-TrCP WT or HA-β-TrCP R474A mutant for 36 h, cell extracts were IP with anti-HA beads, and the immunocomplexes were subjected to western blot with the indicated antibodies

The ARID1A protein levels were rapidly decreased during DNA damage response and this decreased was major caused by proteasome. In gastric cancer NCI-N87 cells, we have found that DNA damage treatment caused significant decrease of ARID1A protein levels which were drastically increased by the administration of MLN4924 or overexpression of a dominant negative Cullin1 mutant [15]. MLN4924 is a potent NEDD8 activating enzyme (NAE) inhibitor and able to inhibit the activity of all the Cullin-based E3 ligases, which need neddylation to fully activation [15]. Thus, these data indicated that ARID1A could be regulated by a SCF complex and recognized by an F-box protein. Then, we screened a panel of FLAG-tagged F-box proteins in HEK293T cells for the binding to endogenous ARID1A. As expected, all of the F-box proteins we screened interacted with SKP1, but β-TrCP was the only one able to bind to ARID1A (Fig. 1a). We further confirmed this interaction in 293 T cells using ectopic expressed exogenous proteins (Fig. 1b). β-TrCP recruits substrates using its WD40 domains and a β-TrCP R474A mutant lost its substrate recognizing activity [21]. As expected, in contrast to β-TRCP wildtype (WT), TRCP R474A mutant failed to interact with ARID1A (Fig. 1c). Together, these data indicated that β-TrCP associated with ARID1A via its WD40 domain.

### β-TrCP controls ARID1A protein levels

**Fig. 2:**
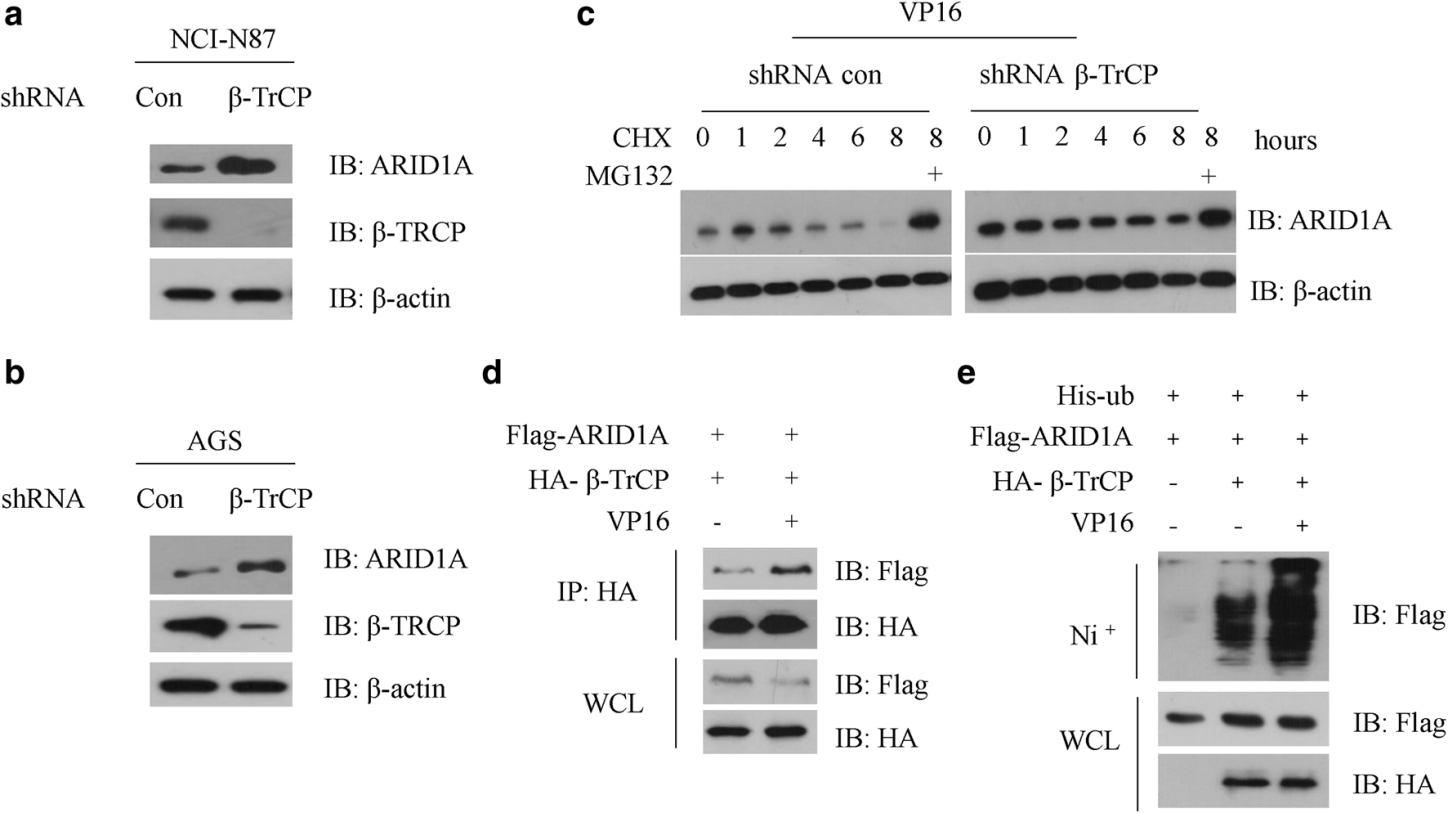
β-TrCP controls ARID1A protein levels. **a** NCI-N87 cells were transfected with con-shRNA or shRNA specific against β-TrCP for 36 h, cell extracts were subjected to western blot with the indicated antibodies. **b** AGS cells were transfected with con-shRNA or shRNA specific against β-TrCP for 36 h, cell extracts were subjected to western blot with the indicated antibodies. **c** NCI-N87 cells were transfected with con-shRNA or shRNA specific against β-TrCP for 24 h, and treated with 2 μg/ml VP16 for 6 h, 25 μg/ml (cycloheximide) CHX was added for the indicated time course. Cell extracts were subjected to western blot with the indicated antibodies. **d** NCI-N87 cells were co-transfected with both Flag-ARID1A and HA-β-TrCP plasmids for 24 h, and then treated with or without 2 μg/ml VP16 for additional 6 h. Cell extracts were IP with anti-HA antibody, and the immunocomplexes were subjected to western blot with the indicated antibodies. **e** NCI-N87 cells were transfected with Flag-ARID1A and his-ubiquitin with or without HA-β-TrCP plasmids for 24 h, and then treated with or without 2 μg/ml VP16 for additional 6 h, 20 μM MG132 was added 4 h before cell harvest. Cell extracts were purified with Ni^+^ beads, and the immunocomplexes were subjected to western blot with the indicated antibodies

To investigate whether β-TrCP affects ARID1A protein abundance, we measured the protein levels of ARID1A upon β-TrCP knockdown (KD) using short hairpin RNAs (shRNAs). Compared to control cells, β-TrCP KD increased ARID1A protein levels in both NCI-N87 and AGS cells, suggesting β-TrCP controls the stability of steady state ARID1A (Fig. 2a, b). As DNA damage reagent VP16 treatment could accelerate the degradation of ARID1A, we then measured the half-life of ARID1A in control or β-TrCP KD AGS cells after DNA damage reagent treatment. We found that VP16 administration significantly decreased the half-life of ARID1A in control AGS cells, but not in β-TrCP KD AGS cells, indicating β-TrCP is required for the destruction of ARID1A during DNA damage response (Fig. 2c). Moreover, DNA damage clearly enhanced the binding of ARID1A to β-TrCP in NCI-N87 cells (Fig. 2d). Finally, we tested whether ARID1A can be ubiquitinated by β-TrCP. Expression of β-TrCP increased ARID1A ubiquitination which could be further enhanced by vp16 treatment (Fig. 2e). Taken together, these data suggesting that β-TrCP controls ARID1A stability in response to DNA damage insult.

### A canonical DSGXXS in ARID1A mediated the interaction between β-TrCP and ARID1A

**Fig. 3:**
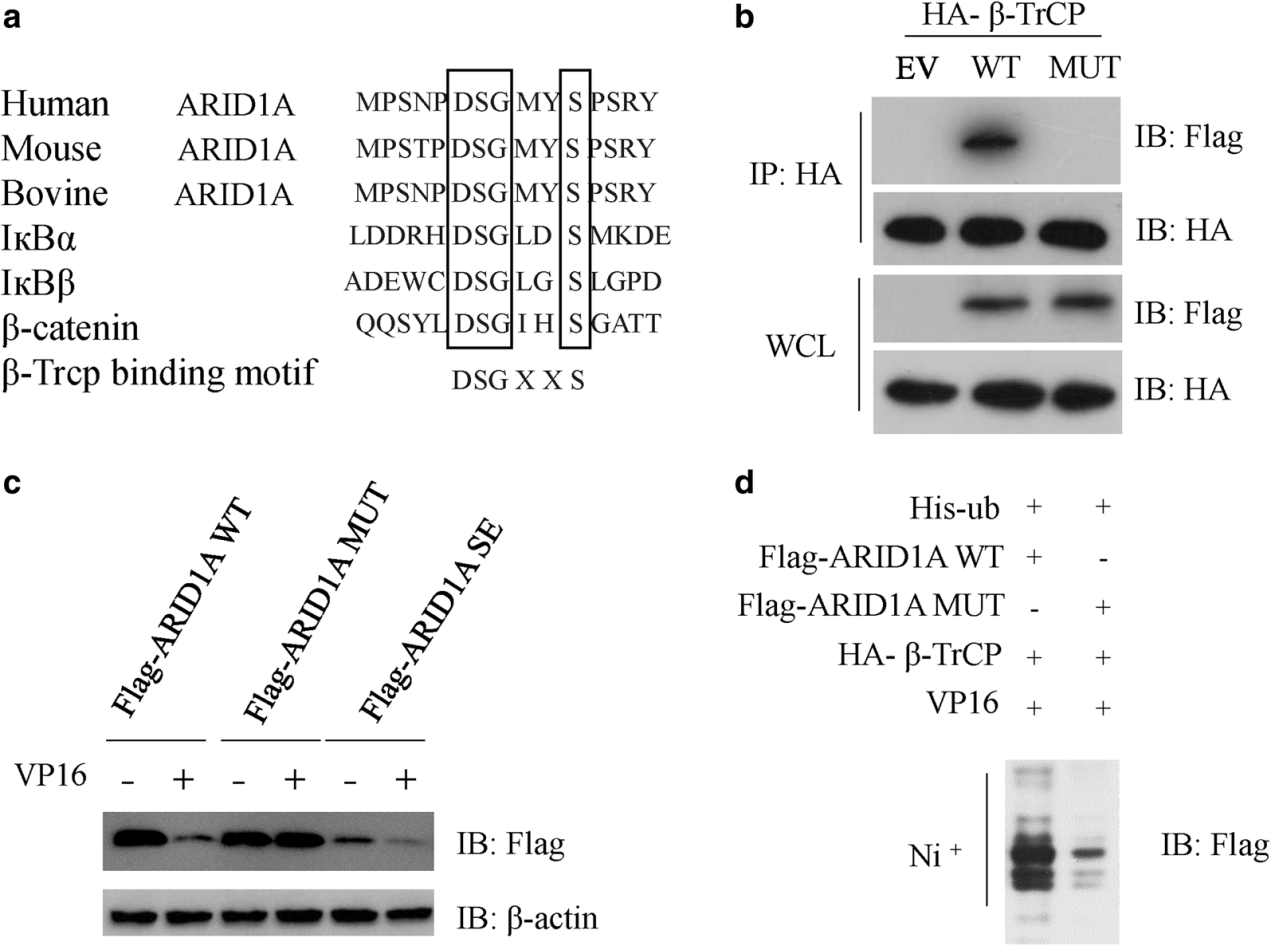
A canonical DSGXXS in ARID1A mediated the interaction between β-TrCP and ARID1A. **a** Alignment of amino acids corresponding to the DSGxxS sequence with ARID1A orthologs and other β-TrCP substrates. **b** HEK293T cells were co-transfected with HA-β-TrCP and Flag-ARID1A WT or Flag-ARID1A SA mutant for 36 h, cell extracts were IP with anti-HA beads, and the immunocomplexes were subjected to western blot with the indicated antibodies. **c** NCI-N87 cells were transfected Flag-ARID1A WT or Flag-ARID1A SA or SE mutants for 24 h, and then treated with or without 2 μg/ml VP16 for additional 12 h. Cell extracts were subjected to western blot with the indicated antibodies. **d** NCI-N87 cells were transfected with HA-β-TrCP, his-ubiquitin with Flag-ARID1A WT or MUT plasmids for 24 h, and then treated with 2 μg/ml VP16 for additional 6 h, 20 μM MG132 was added 4 h before cell harvest. Cell extracts were purified with Ni^+^ beads, and the immunocomplexes were subjected to western blot with the indicated antibodies

β-TrCP recognizes two phosphorylated serine residues in a DSGXXS sequence in its substrates [22]. By searching the protein sequence, we found that ARID1A contains a cross-species conserved β-TrCP canonical recognition sequence (DSGMYS) (Fig. 3a). Next, we asked whether the DSGMYS residues are sufficient for β-TrCP binding. To this end, we generated a mutant in which both serine residues were mutated to alanine (S1316A, S1320A, ARID1A SA mutant). We then test its binding activity to β-TrCP. In contrast to ARID1A WT, the ARID1A SA mutant failed to interact with β-TrCP, suggesting the phosphorylation of serine residues within DSGMYS is required for the binding to β-TrCP (Fig. 3b). We also generated another mutant in which both serine residues were mutated to glutamic acid (S1316E, S1320E, ARID1A SE). As expected, VP16 treatment failed to decrease the protein levels of ARID1A SA mutant (Fig. 3c). However, ARID1A SE protein was less stable when compared with the WT/MUT proteins with or without DNA damage insult (Fig. 3c). In concert with this, DNA damage insult also failed to enhanced the ubiquitination of ARID1A SA mutant (Fig. 3d). Overall, the data demonstrate that β-TrCP initiates degradation of ARID1A after recognition of a DSGXXS motif in which the residues S1316 and S1320 are need to be phosphorylated.

### ATM-induced ARID1A phosphorylation promoted β-TrCP-induced ARID1A destruction

**Fig. 4:**
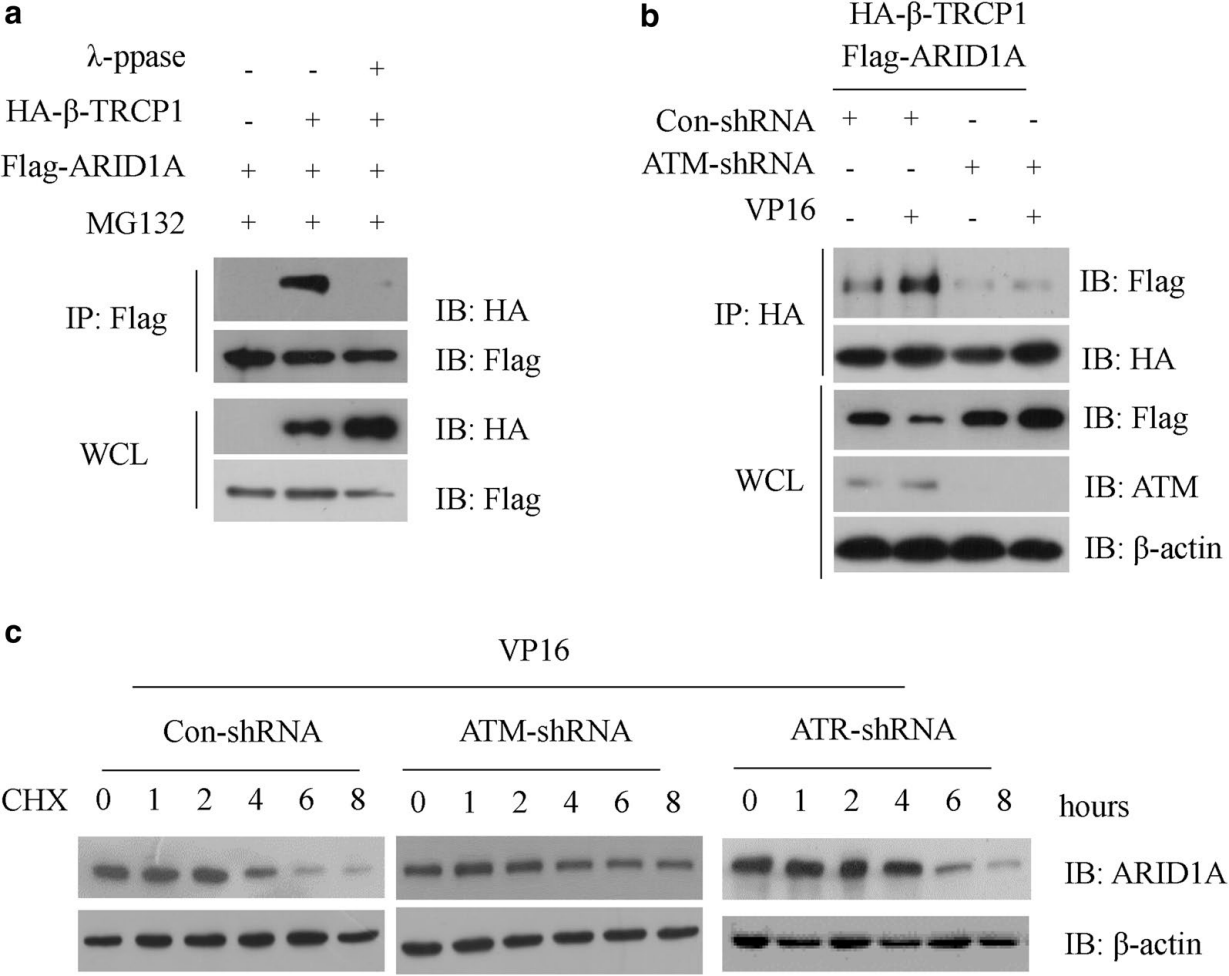
ATM-induced ARID1A phosphorylation promoted β-TrCP-induced ARID1A destruction. **a** NCI-N87 cells were co-transfected with Flag-ARID1A and HA-β-TrCP for 36 h, 20 μM MG132 was added for additional 6 h. Cell extracts were treated with λ-PPase for 1 h at 30 °C and IP with anti-HA beads. The immunocomplexes were subjected to western blot with the indicated antibodies. **b** NCI-N87 cells were transfected with con-shRNA or shRNA specific against ATM for 24 h and then treated with or without 2 μg/ml VP16 for additional 6 h, cell extracts were IP with anti-Flag M2 beads, and the immunocomplexes were subjected to western blot with the indicated antibodies. **c** NCI-N87 cells were transfected with con-shRNA or shRNA specific against ATM or ATR for 24 h and then treated with or without 2 μg/ml VP16 for additional 6 h, 25 μg/ml (cycloheximide) CHX was added for the indicated time course. Cell extracts were subjected to western blot with the indicated antibodies

In consistent with the above-mentioned data, we found that λ-ppase treatment significantly prevented the interaction between β-TrCP and ARID1A (Fig. 4a), further confirming the requirement of ARID1A phosphorylation for β-TrCP recognition. We then asked which kinase is involved in this process. DNA breaks induce a coordinated set of molecular signaling events which leading to activate a nuclear kinase ataxiatelangiectasia mutated (ATM) [23]. The ATM kinase activity is essential for most cellular signaling in response to DNA damage treatment [24]. We hypothesis that ATM might be involved in the degradation of ARID1A. Indeed, DNA damage-induced ARID1A destruction is absent in ATM knockdown NCI-N87 cells. Then, we performed Co-IP experiments of ARID1A and β-TrCP with or without DNA damage in ATM WT or KD cells and found that the interaction between ARID1A and β-TrCP was enhanced by DNA damage and ATM kinase deficiency could reverse this phenomenon (Fig. 4b). Moreover, the endogenous ARID1A protein was more stable in ATM KD cells during DNA damage response when compared with its WT counterpart, but not in ATR KD cells (Fig. 4c). Thus, these data indicated that ATM kinase is required for the destruction of ARID1A during DNA damage response.

## Discussion

By epigenetically regulating gene expression, the SWI/SNF chromatin remodeling complex mediates diverse biological pathways including DNA damage response [25]. ARID1A, a component of the SWI/SNF complex, is a tumor suppressor with a high frequency of inactivating mutations in many cancers and plays an important role in targeting the complex to gene promoters [25]. ARID1A and the SWI/SNF complex are capable of activating or repressing the transcription of hundreds of target genes. For example, p21 (CDKN1A) has been identified as a target gene of ARID1A and mediated the growth-suppressive effects of ARID1A [26].

In the present study, our data reveal a novel molecular event taking place in DNA damage response which is mediated by ATM and β-TrCP. Our previous studies suggest that ARID1A was associated with a SCF complex. Thus, we focus on identification the exact F-box protein which is responsible for the recognizing and destruction of ARID1A. By using unbiased F-box protein library screen, we identify that β-TrCP controls the stability of ARID1A. Our biochemical data reveal that ARID1A is a novel substrate of β-TrCP which interacts and targets phosphorylated ARID1A for ubiquitination and degradation. We show that ARID1A is recognized by β-TrCP through a DSGXXS motif after phosphorylation of two serine residues (S1316 and S1320). Although we have not identified the kinase directly responsible for phosphorylation of residues S1316 and S1320, we still able to provide evidence to show that the DNA damage-activated kinase ATM is involved in the degradation of ARID1A. As genetic or pharmacologic inactivation of the Ataxia Telangiectasia Mutated (ATM) kinase impaired DNA damage-induced ARID1A destruction. However, the in vitro kinase assay should be utilized in the future to clarify whether ATM is the direct kinase or the upstream kinase. The phosphorylation of ARID1A would ensure rapid degradation of ARID1A to transcriptional activate or repress the expression of some genes needs for DNA damage checkpoint activation and preventing the cell death, as we previously have found that overexpression of ARID1A protein caused significant cell death after DNA damage insult in gastric cancer cells.

## Conclusions

By using unbiased F-box protein library screen, our study is the first to show F box protein β-TrCP controls the stability of ARID1A. We further found that ARID1A is recognized and ubiquitinated by β-TrCP through a DSGXXS motif after phosphorylation of two serine residues (S1316 and S1320) during DNA damage response. We provide a novel molecular mechanism for the negative regulation of ARID1A by β-TRCP and ATM in gastric cancer cells in response to DNA damage insult. Our data suggest that ARID1A acts as a participant in the damage response pathway, the fine tune of which might contribute to tumorigenesis prevention and affect the response of patients towards DNA-damaging chemotherapies.

## Data Availability

Not applicable.

## Abbreviations

ARID1A: AT-rich interactive domain-containing protein 1A
GWAS: genome-wide association studies
SWI/SNF: switch/sucrose non-fermentable
GC: gastric cancer
SCF: Skp1-Cul1-F box protein
CHX: cycloheximide
NC: nitrocellulose
HRP: horseradish peroxidase
KD: knockdown
shRNAs: short hairpin RNAs
ATM: Ataxia Telangiectasia Mutated
IP: immunoprecipitation
